# Characterization of the Hydrogel-Binding Mechanism of *Phellinus linteus* Polysaccharides and Polymerized Whey Protein by SR-IR Technology and Its Application in Goat Milk Yogurt

**DOI:** 10.3390/foods15040699

**Published:** 2026-02-13

**Authors:** Qingyun Wang, Xingyu Zhang, Yilong Li, Huiyu Xiang, Weibing Tao, Jiafu Wu, Jiping Fan, Huangchen Xi, Lin Wang, Juncai Hou, Xiaomeng Sun

**Affiliations:** 1 Key Laboratory of Dairy Science, College of Food Science, Northeast Agricultural University, Harbin 150030, China; wangshuaifood@163.com (Q.W.); 15930315145@163.com (X.Z.); liyilongdyx@163.com (Y.L.); 13534253985@163.com (H.X.); t1548637592@163.com (W.T.); j272513@126.com (J.W.); fjp215@126.com (J.F.); xihuangchen@outlook.com (H.X.); wswlwlsw2022@163.com (L.W.); 2 Beidahuang Wandashan Dairy Co., Ltd., Harbin 150078, China; 3 College of Food Science and Engineering, Guiyang University, Guiyang 550005, China; 4 Danisco (China) Co., Ltd., Kunshan 215300, China

**Keywords:** *Phellinus linteus* polysaccharides, polymerized whey protein, goat yogurt, texture

## Abstract

The development of natural biopolymers to improve the structural and textural defects of whey protein in acidic dairy products is of great interest. This study investigated the interaction between *Phellinus linteus* polysaccharides (PLPs) and heat-induced polymerized whey protein (PWP), and its application in goat milk yogurt. The physicochemical properties and interaction mechanisms of PWP-PLP composite hydrogels (with 1–4% PLP) were characterized using particle size, DSC, and synchronous rheology and Fourier transform infrared spectroscopy. The results show that PLP increased hydrogel particle size, absolute zeta potential (from −34.98 mV to −42.26 mV), and denaturation temperature (from 102.33 °C to 112.57 °C), indicating the enhanced stability. SR-IR analysis revealed intensified hydrogen bonding and protein secondary structure rearrangement. Incorporated into yogurt, the optimal composite (10% PWP with 3% PLP) significantly improved water-holding capacity (by 20–30%), storage modulus (G′), and produced a denser microstructure with superior texture. This work demonstrates that PLP is an effective natural modifier for constructing stable protein–polysaccharide hydrogels to enhance yogurt quality.

## 1. Introduction

Yogurt, an important fermented dairy product, combines desirable flavor, a thick and creamy texture, and high nutritional density, making it a valuable source of high-quality dietary proteins [1]. Its main protein components, whey proteins and caseins, are rich in essential amino acids and exhibit excellent digestibility and absorption efficiency, thereby meeting the protein supply needs of specific populations [2]. In addition, the fine and uniform hydrogel structure of yogurt provides unique benefits for individuals with dysphagia. To further enhance the nutritional density and textural properties of yogurt, whey protein isolate (WPI) is commonly incorporated as both a thickening agent and a protein-fortifying ingredient. WPI is rich in β-lactoglobulin and α-lactalbumin, and exhibits excellent emulsifying and hydrogelling properties [3], as well as high biological value. However, under acidic conditions or thermal processing, WPI is prone to irreversible aggregation, resulting in the formation of coarse hydrogel networks that negatively affect mouthfeel, reduce water-holding capacity, and promote whey separation [4,5]. In addition, the partial hydrolysis products of WPI may impart bitterness [6], further limiting its use in high-quality structured foods, particularly those intended for individuals with dysphagia. Therefore, the development of natural biopolymers capable of improving WPI hydrogel structure, enhancing sensory quality, and increasing processing stability is of significant importance.

Protein–polysaccharide composite hydrogels have gained increasing attention in food science in recent years due to their controllable structural properties, favorable stability, and natural biodegradability. Polysaccharides can interact with proteins through electrostatic interactions, hydrogen bonding, and hydrophobic forces to form stable complexes, thereby enhancing hydrogel strength, elasticity, thermal stability, and water-holding capacity, while reducing protein aggregation-induced coarse structures and improving rheological behavior [7]. Numerous studies have demonstrated that plant-derived polysaccharides, *Mesona chinensis* polysaccharide, *Curdlan*, and *Fucoidan* polysaccharide, can promote the formation of more uniform three-dimensional networks in WPI hydrogels, increase the β-sheet content, and accelerate gelation kinetics. These findings highlight the significant synergistic effects of polysaccharides in modulating the structural and functional properties of protein-based hydrogels [8,9,10].

*Phellinus linteus* polysaccharide (PLP), extracted from the medicinal fungus *Phellinus linteus*, consists primarily of β-glucans and various heteropolysaccharide complexes and exhibits multiple biological activities [11], including immunomodulatory, anti-inflammatory, antioxidant, and metabolic regulatory effects [12]. These properties highlight its considerable potential as an active ingredient in functional food applications. Despite the extensive research on the bioactivities of PLP, the structural interaction mechanisms between PLP and WPI or thermally induced polymeric whey protein (PWP) remain incompletely understood. In particular, limited information is available regarding their compatibility within composite hydrogel systems, the resulting network assembly, interfacial behaviors, and the synergistic effects on the macroscopic properties of the hydrogels. A deeper understanding of the interaction mechanisms between PLP and PWP would not only enable the design of protein–polysaccharide hydrogel systems with finely tunable structures but also facilitate the development of functional thickening agents and medical foods for specialized nutritional needs [13]. Critically, the functional enhancements observed in model hydrogel systems are directly transferable to complex food matrices like yogurt. They may improve the physical and chemical properties and sensory characteristics of yogurt [14]. A mechanistic understanding of how PLP modulates PWP aggregation and network formation at the molecular level provides a rational basis for formulating yogurt with tailored textural and stability attributes. By first elucidating the PLP-PWP interaction mechanism in a controlled hydrogel system, we can strategically select an optimized composite with the most desirable functional profile for subsequent incorporation into yogurt, thereby bridging fundamental insight with practical application.

Simultaneous rheology and Fourier transform infrared spectroscopy (SR-IR) is a novel analytical technique that combines rheological measurements with real-time Fourier transform infrared spectroscopy [15,16]. Compared with traditional separate measurement methods, SR-IR can simultaneously obtain the macroscopic rheological behavior and the microscopic molecular structural changes in the sample during heating or shearing under the same experimental conditions, thereby enabling direct correlation and dynamic analysis of the structure–property relationship [17,18]. This study employed the SR-IR technique to elucidate the cooperative gelation behavior of PWP and PLP during the heat-induced process. From the perspective of structure–function correlation, it revealed the gelation enhancement mechanism.

Building on the above considerations, this study hypothesizes that PLP can interact with PWP, thereby enhancing hydrogel’s strength, thermal stability, and water-holding capacity while mitigating the brittleness and syneresis typical of WPI hydrogels, and adding a PWP-PLP hydrogel to goat milk yogurt can significantly improve the properties of yogurt. To test this hypothesis, rheology and SR-IR were employed to elucidate the structural characteristics and interaction mechanisms of PWP-PLP hydrogels prepared with varying PLP levels, complemented by low-temperature SEM to resolve microstructural assembly. The optimized hydrogels were subsequently incorporated into a goat milk yogurt system to evaluate their effects on texture, physicochemical properties, and microstructure. Overall, this work aimed to develop a natural protein–polysaccharide thickening system with combined nutritional, textural, and functional benefits, providing a promising strategy for specialized medical foods and broadening the potential applications of PWP-PLP hydrogels in food processing and biomedical materials.

## 2. Materials and Methods

### 2.1. Materials

Whey protein isolate (WPI) with a purity of 93.14% was acquired from Fonterra Co., Ltd. (Auckland, New Zealand). *Phellinus linteus* polysaccharide (PLP) at 80% purity was sourced from Peimei Biotechnology Co., Ltd. in Xi’an, China (formerly referred to as Time Biology Co., Ltd.). 8-Anilinonaphthalene-1-sulfonic acid (ANS) and phosphate-buffered saline (PBS) were procured from the Sigma-Aldrich Corporation (St. Louis, MO, USA). 5,5′-Dithiobis (2-nitrobenzoic acid) (DTNB) was supplied by Maclin Biochemical Technology Co., Ltd. (Shanghai, China). Tris (hydroxymethyl) aminomethane (Tris), glycine (Gly), and ethylenediaminetetraacetic acid (EDTA) were obtained from Thermo Fisher Scientific Co., Ltd. (Waltham, MA, USA). The 2,2-diphenyl-1-picrylhydrazyl (DPPH) reagent was purchased from Beijing Bio-Ceramic Technology Co., Ltd. (Beijing, China). The starter culture ABY-8, which consists of *Streptococcus thermophilus* and *Lactobacillus delbrueckii* subsp. Bulgaricus was obtained from Chr. Hansen A/S (Hørsholm, Denmark). Goat milk powder was purchased from Beijing Zhongtian Xuteng Food Co., Ltd. (Beijing, China). All chemical reagents utilized in the present study met analytical grade standards.

### 2.2. Preparation of PWP-PLP Composite Hydrogel

#### 2.2.1. Preparation of Stock Solutions

A 20% (*w*/*v*) stock solution of whey protein isolate (WPI) was prepared by dispersing the corresponding WPI powder in deionized water. The mixture was then stirred continuously at 400 rpm for 3 h at ambient temperature using a CMAG HS7 magnetic stirrer (IKA, Staufen, Germany) to ensure complete dissolution. For the *Phellinus linteus* polysaccharide (PLP) stock solution, PLP powder was dissolved in deionized water to achieve a final concentration of 8% (*w*/*v*), with stirring conducted at 700 rpm for 0.5 h under the same room temperature conditions. After initial preparation, all stock solutions were transferred to a refrigerator set at 4 °C for overnight hydration to facilitate full molecular dispersion.

#### 2.2.2. Preparation of PWP-PLP Hydrogels

WPI and PLP stock solutions were mixed at room temperature to obtain final levels of 10% (*w*/*v*) WPI and 1%, 2%, 3%, and 4% (*w*/*v*) PLP. The final volume of each sample was adjusted to 50 mL with deionized water. A 10% (*w*/*v*) WPI solution without PLP served as the control. The pH of each mixture was adjusted to 7.0 using 1 M NaOH, followed by heating in a water bath at 85 °C for 30 min under continuous stirring. After heating, the samples were cooled in an ice-water bath to obtain the PWP-PLP hydrogels, designated PLP1 to PLP4, while the control PWP hydrogel without PLP was designated PLP0.

### 2.3. Particle Size and Zeta Potential Measurement

Particle size and zeta potential were measured following the method of Zhang et al. [14] with minor modifications. Measurements were performed at 25 °C using a Nano ZS Zetasizer (Malvern Instruments, Malvern, UK). PWP-PLP hydrogels prepared with different PLP levels were diluted 100-fold with deionized water before analysis. Each sample was measured in triplicate, and the average value is reported.

### 2.4. The Differential Scanning Calorimetry (DSC) Measurement

Based on a protocol with minor modifications inspired by the work of Zhang et al. [14], the thermal properties of PWP-PLP hydrogels were characterized using a differential scanning calorimeter (DSC3, Mettler Toledo, Zurich, Switzerland). Before DSC analysis, all hydrogel samples were freeze-dried using a Martin Christ Alpha 1-2 freeze-dryer (Osterode, Germany) to remove moisture interference. For each test, approximately 5 mg of the lyophilized sample was accurately weighed and placed into an aluminum pan, which was then hermetically sealed. The sample pan was heated from 30 °C to 200 °C at a constant heating rate of 10 °C per min. To eliminate background thermal effects from the instrument or environment, a sealed empty aluminum pan was used as the reference control throughout the entire thermal scanning process.

### 2.5. The Rheological Measurements

#### 2.5.1. The Apparent Viscosity Measurement

The apparent viscosity was measured using a rotational MARS40 dynamic shear rheometer (Thermo Fisher Scientific, Karlsruhe, Germany) equipped with a 30 mm rotor and a 60 mm plate, following the method described by Lv et al. [19] with slight modifications. A total of 10 mL of the PWP-PLP hydrogel sample was loaded onto the rheometer, and the shear rate was controlled within the range of 10–600 s^−1^. The apparent viscosity was recorded at a shear rate of 100 s^−1^.

#### 2.5.2. The Dynamic Rheological Measurement

Dynamic rheological characterization of the as-prepared hydrogels was carried out with minor modifications based on the protocol described by Luo et al. [20]. A parallel-plate configuration was adopted for the measurements, consisting of a 35 mm upper plate and a 60 mm lower plate, with the gap between the plates precisely adjusted to 0.8 mm. To minimize moisture evaporation during the thermal treatment process, silicone oil was uniformly applied around the edges of the hydrogel samples. The thermal cycle for the samples involved heating from 25 °C to 85 °C at a constant rate of 5 °C per min, maintaining isothermal conditions at 85 °C for 30 min, and subsequently cooling back to 25 °C at a rate of 1 °C per min. All rheological tests were performed at a fixed strain amplitude of 0.1%, which was confirmed to be within the linear viscoelastic region of the hydrogels to avoid structural damage. At 85 °C, the storage modulus (G′) and loss modulus (G″) of the samples were recorded as functions of angular frequency, with the frequency range spanning from 0.1 Hz to 25 Hz.

### 2.6. Two-Dimensional (2D) and Three-Dimensional (3D) Intrinsic Fluorescence Spectroscopy

The intrinsic fluorescence spectra of the PWP-PLP hydrogels were recorded using an F-4500 fluorescence spectrophotometer (Hitachi, Tokyo, Japan). The procedure was performed according to a previously reported method with slight modifications [21]. Before measurement, the samples were diluted 1000-fold with deionized water. The 2D intrinsic fluorescence spectra were collected at an excitation wavelength of 280 nm, with emissions scanned from 290 to 300 nm, using a slit width of 5 nm. The 3D intrinsic fluorescence was collected at an excitation wavelength of 260–300 nm, with emission wavelengths of 200–500 nm, using a slit width of 5 nm [7].

### 2.7. The Surface Hydrophobicity Measurement

Fluorescence spectroscopy was performed using an F-4500 fluorescence spectrophotometer (Hitachi, Tokyo, Japan) following the methods reported by Zhang et al. [22], with minor modifications. A total of 20 μL of ANS solution (8 mM) was added to 4 mL of the diluted sample solution (protein level: 10 mg/mL), and the mixture was incubated in the dark for 15 min. Fluorescence spectra were recorded at an excitation wavelength of 370 nm, with emissions collected from 400 to 600 nm.

### 2.8. The Free Sulfhydryl Content Measurement

The sulfhydryl content was determined using the Ellman method, following the procedures described by Zhang et al. [14] and Wang et al. [23] with minor modifications. The protein solution (1 mg/mL) was diluted with Tris–Gly buffer (0.086 M Tris, 0.09 M glycine, and 0.004 M EDTA; pH 8.0). Subsequently, 50 μL of Ellman’s reagent was added to the diluted protein solution, and the mixture was incubated in the dark for 30 min. The absorbance was measured at 412 nm using Tris–Gly buffer as the blank. The *SH* content was calculated according to the following equation:

*SH* (μmol/g) = 73.53 × *A*_412_ × *D*/*C* (1)

In this formula, *A*_412_ refers to the absorbance value of the sample measured at a wavelength of 412 nm. *D* stands for the dilution factor applied during sample pretreatment, and *C* represents the protein concentration in the tested solution. The constant 13,600 corresponds to the molar extinction coefficient specific to the Ellman reaction, which is used to convert the measured absorbance data into the quantitative value of sulfhydryl groups in the sample.

### 2.9. Synchronous Rheology (SR-IR) and Fourier Transform Infrared Spectroscopy (FT-IR)

FTIR was used to monitor the structural changes occurring during the formation of PWP-PLP hydrogels. The measurements were performed according to the method described by Zhu et al. [24] with minor modifications, using a rheo-FTIR system consisting of a HAAKE MARS 40 rheometer (Thermo Fisher Scientific, Waltham, MA, USA) equipped with a Nicolet iS10 FTIR spectrometer (Thermo Fisher Scientific, Waltham, MA, USA). Samples were prepared as described in Section 2.4. Dynamic rheological conditions were applied during FTIR acquisition. The FTIR spectra were recorded over the range of 4000–400 cm^−1^ with a scanning rate of two scans per second.

### 2.10. Molecular Docking Analysis

Molecular docking represents a robust computational validation technique for the evaluation of binding affinities between small molecules. In this study, a semi-flexible docking approach was employed using AutoDock Vina (version 1.1.2) [19]. WPI is a complex mixture of proteins, with β-lactoglobulin (β-Lg) and α-lactalbumin (α-La) being the predominant components. This study did not involve a direct experimental comparison between specific WPI fractions, as in previous research [25]. This research used molecular docking simulations, which were conducted using the major whey proteins (β-Lg and α-La) to model their interactions with PLP oligosaccharides. This approach provides mechanistic insights at the molecular level and complements the macroscopic experimental findings. The three-dimensional structures of α-lactalbumin and β-lactoglobulin were obtained from the RCSB Protein Data Bank (https://www.pdb.org/ (accessed on 29 January 2026)) [26,27], while the structure of PLP was acquired from the PubChem database (https://pubchem.ncbi.nlm.nih.gov/ (accessed on 29 January 2026)). Protein structures were prepared using AutoDockTools (http://mgltools.scripps.edu/downloads (accessed on 29 January 2026)); this involved the addition of hydrogen atoms, assignment of partial charges, definition of atom types according to the AutoDock 4 force field, and generation of a docking grid box encompassing the relevant binding site. Ligand structures were similarly processed by defining the root and identifying rotatable bonds. Both receptor and ligand files were subsequently converted from the standard PDB format to the requisite PDBQT format using AutoDockTools to enable docking calculations. Following the execution of molecular docking with AutoDock Vina, the resulting binding poses were scored to estimate affinity. A detailed analysis of the intermolecular interactions within the predicted complexes was conducted. Visualization and further examination of the docking outcomes were performed using PyMOL (version 2.6.2) and Discovery Studio 2019 software.

### 2.11. Two-Dimensional Correlation Spectroscopy Technology

Data processing and analysis were conducted using Origin2024 software [19].

### 2.12. Preparation of Yogurt

Goat milk powder was mixed with sucrose at a ratio of 7% (*w*/*v*), and the mixture was reconstituted with deionized water to the desired level, ensuring complete dissolution. The mixture was pasteurized at 85 °C for 30 min in a thermostatic water bath. After cooling to 43 °C, 0.03% (*w*/*v*) ABY-8 starter culture was added, followed by the incorporation of PLP (1.5%, 3%, 4.5%, or 6%) or PWP-PLP hydrogels containing 10% PWP. The inoculated samples were incubated at 43 °C for approximately 6 h until the pH dropped below 4.5. Upon completion of fermentation, the yogurt samples were cooled and stored at 4 °C for maturation prior to subsequent analyses [24].

### 2.13. pH Value of Yogurt

The pH of the yogurt was measured every 45 min using a pH meter (PHSJ-3C, INESA, Shanghai, China) [28,29].

### 2.14. Yogurt Particle Size

The particle size distribution was analyzed using a laser diffraction particle analyzer (Mastersizer 3000E, Malvern, UK). Samples were dispersed in deionized water until the obscuration reached approximately 50% [29].

### 2.15. Water-Holding Capacity (WHC) of Yogurt

*WHC* of the samples was determined according to the method described by Lv et al. [19] with slight modifications. Before fermentation, the samples were accurately weighed (*m*_1_) and transferred into pre-weighed centrifuge tubes (*m*_0_). The samples were fermented at 43 °C for 6 h and subsequently stored at 4 °C for 24 h for maturation. The tubes were then centrifuged at 4000 rpm for 20 min, after which the supernatant was removed and the precipitate was weighed (*m*_2_). The WHC was calculated according to the following equation:

(2) $$WHC\left(\%\right)=\frac{m_{2}-m_{0}}{m_{1}-m_{0}}\times 100$$

### 2.16. Rheological Properties of Yogurt

The measurements were conducted using a Rheonaut rheometer (Thermo Scientific HAAKE MARS 40, Waltham, MA, USA), adhering to a previously reported experimental protocol with slight adaptations to match the specific requirements of the present study [20]. To ensure the reliability and reproducibility of the data, each sample was tested in triplicate, and the final results were expressed as the mean ± standard deviation.

### 2.17. Scanning Electron Microscopy of Yogurt

The microstructure of the yogurt samples was examined using a scanning electron microscope (SEM, SU8010, Hitachi, Japan). Prior to observation, the samples were cryo-fixed in liquid nitrogen and subsequently freeze-dried to obtain solid specimens. These specimens were then sputter-coated with a thin gold layer to enhance conductivity before being imaged under a specified acceleration voltage [19].

### 2.18. Texture Measurement of Yogurt

Texture profile analysis (TPA) was performed using a texture analyzer (TA.XTplusC, Stable Micro Systems, Surrey, UK) equipped with an A/BE-d35 cylindrical probe. The target deformation was set to 25 mm, with a test speed of 1.0 mm/s and a trigger force of 5 g. Each sample was measured in triplicate. The texture parameters of yogurt were analyzed using the instrument’s software, following the method described by Zhou et al. [30].

### 2.19. Statistical Analysis

All experiments were carried out in triplicate to ensure data reliability, and the results were presented as mean ± standard deviation (SD). Statistical analysis was performed using Origin software, where one-way analysis of variance (ANOVA) was first conducted to evaluate overall differences among groups, followed by Tukey’s post hoc test for pairwise comparisons. A *p*-value less than 0.05 was considered to indicate a statistically significant difference [14].

## 3. Results and Discussion

### 3.1. Dynamic Rheology Analysis

**Figure 1:**
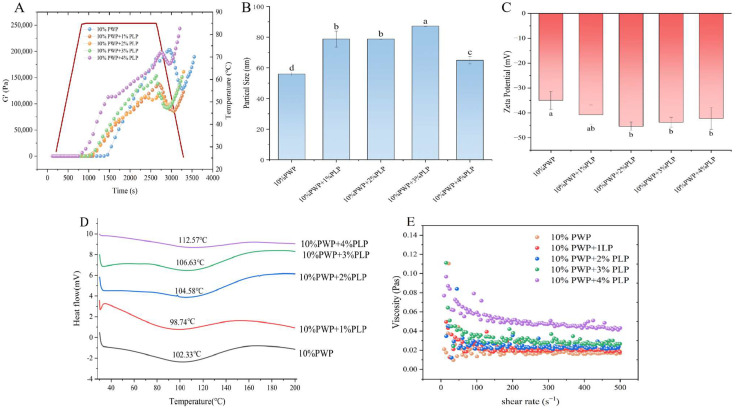
Dynamic rheology (**A**), particle size (**B**), zeta potential (**C**), DSC (**D**), and surface viscosity (**E**) of PWP-PLP with 10% PWP and different PLP levels (0%, 1%, 2%, 3%, and 4% *w*/*v*).

The G′ reflected the solid-like behavior of hydrogels, and changes in G′ during heat-induced gelation indicate the development of the hydrogel network [31]. According to Ban et al. [32], when G′ exceeded 1 Pa and exhibited the maximum rate of increase, a three-dimensional hydrogel structure was considered to have formed. In this study, the effects of different PLP levels (1–4%) on the gelation behavior of 10% PWP were compared by monitoring the evolution of G′ as a function of temperature and time (Figure 1A) (Red line denotes gelation temperature). As the level of PLP increased, the gelation time of the PWP-PLP hydrogel decreased from 1190 s (PLP0) to 1013 s, 920.6 s, 893.3 s, and 840.3 s, respectively. Meanwhile, G′ values consistently increased with rising temperature, indicating the progressive formation and reinforcement of the hydrogel network. The accelerated gelation process and enhanced G′ in the presence of higher PLP levels might be attributed to the unfolding of PLP molecular chains and the strengthening of intermolecular interactions during heating. These interactions likely involving hydrogen bonding, hydrophobic associations, and potential electrostatic attraction, facilitate the formation of a more cohesive 3D network, thereby improving the gelation characteristics of the PWP-PLP hydrogel [33,34].

### 3.2. Particle Size Analysis of PWP-PLP Hydrogels

Particle size is a key indicator reflecting the aggregation behavior and stability of hydrogel systems [14]. The particle sizes of 10% PWP hydrogel and PWP-PLP hydrogels containing different PLP levels (1%, 2%, 3%, and 4%) were measured, and the results are shown in Figure 1B (Different letters indicate significant differences). The average particle size of the 10% PWP hydrogel was 55.98 ± 1.08 nm. As the PLP level increased from 0% to 2%, the particle size progressively increased from 55.98 nm to 64.98 nm (+116%). This trend was consistent with the findings of Liu et al. [35], who reported that interactions between *okra* polysaccharides and PWP promoted molecular aggregation and the formation of larger complexes.

### 3.3. Zeta Potential Analysis of PWP-PLP Hydrogel

Zeta potential is commonly used to evaluate the stability of colloidal systems and the surface charge characteristics of dispersed particles [14]. Generally, an absolute zeta potential value greater than 30 mV indicated good colloidal stability [36]. In this study (Figure 1C) (Different letters indicate significant differences), the zeta potential of the 10% PWP hydrogel was −34.98 ± 1.61 mV, suggesting that the PWP hydrogel itself possessed a relatively stable dispersion. Upon the incorporation of PLP, the absolute zeta potential values of all composite hydrogels increased, indicating the enhanced stability of the PWP-PLP system. This improvement might be attributed to the negative charges carried by PLP, which could alter the surface charge density of PWP upon interaction [20]. As the PLP level increased, the introduction of additional negative charged further strengthened electrostatic repulsion among particles, thereby improving hydrogel stability [8]. It was consistent with those of the DLVO theory, which states that the stability of polysaccharide-based hydrogels is governed by the balance between attractive van der Waals forces and repulsive electrostatic interactions. Van der Waals attraction tends to promote particle aggregation, leading to precipitation and consequently compromising hydrogel stability [37]. In contrast, PLPs contained abundant anionic groups that could adsorb onto the protein surface, resulting in a higher absolute value of the zeta potential in the PWP-PLP hydrogel system. The increased surface charge enhanced electrostatic repulsion between particles, effectively counteracting the inherent van der Waals attraction. As a result, electrostatic repulsion became the dominant interaction within the system, preventing particles from approaching each other and aggregating. This enabled the particles to remain uniformly dispersed within the hydrogel network, thereby significantly improving the structural stability of the PWP-PLP hydrogel. Such electrostatic repulsion-dominated stabilization mechanisms have been widely reported in previous studies on protein–polysaccharide hydrogel and colloidal systems [38,39].

### 3.4. Differential Scanning Calorimetry (DSC) Analysis of PWP-PLP Hydrogels

DSC is an effective tool for elucidating the interaction patterns between proteins and polysaccharides and for evaluating the thermal stability of the resulting complexes [29]. In this study (Figure 1D), the addition of PLP induced pronounced changes in the endothermic peak temperatures of the hydrogels. The observed endothermic peaks signify a heat-absorption process and the endothermic peak temperatures of PLP1, PLP2, PLP3, and PLP4 were 101.53 °C, 105.13 °C, 107.15 °C, and 112.34 °C, respectively. The progressive increases in thermal transition temperature clearly indicate that PLP markedly enhances the thermal stability of PWP hydrogels. This improvement might be attributed to the formation of additional hydrogen bonds and hydrophobic interactions between PWP and PLP molecules, which strengthen the hydrogel network and render it more resistant to heat-induced denaturation. Moreover, the incorporation of PLP might alter the spatial conformation of PWP. During heating, such conformational changes could promote protein aggregation and facilitate the development of a more compact and thermally stable structure [9,40]. Overall, the DSC results demonstrate that PLP played a significant role in reinforcing the thermal stability of the PWP-based hydrogel, further supporting the potential of PLP as a functional polysaccharide for improving protein hydrogel systems.

### 3.5. Apparent Viscosity

The apparent viscosity of the PWP-PLP hydrogels increased in a level-dependent manner with the addition of PLP (Figure 1E). The PLP0 sample exhibited relatively low viscosity and showed typical shear-thinning behavior characteristic of protein-based hydrogels, in which viscosity decreased with increasing shear rate due to the disruption of weak intermolecular networks [10,41]. In contrast, the incorporation of PLP markedly enhanced the apparent viscosity across the tested shear rates. A moderate increase was observed for PLP1, while PLP4 displayed the highest viscosity and a more gradual decline at higher shear rates, indicating pronounced shear-thinning behavior. This level-dependent enhancement could be attributed to the synergistic interactions between PWP and PLP. Previous studies had reported that hydrogelling polysaccharides—curdlan and chia seed gum—could increase hydrogel viscosity by forming hydrogen bonds and electrostatic interactions with protein molecules, thereby increasing network density and restricting molecular mobility [42]. The hydroxyl and carboxyl groups in PLP might interact with the amino and sulfhydryl groups of PWP, promoting cross-linking and contributing to the formation of a more compact hydrogel network [9]. At higher PLP levels, the polysaccharide chains might act as additional cross-linking junctions, enhancing resistance to shear deformation and resulting in increased apparent viscosity [35].

### 3.6. Intrinsic Fluorescence Analysis

**Figure 2:**
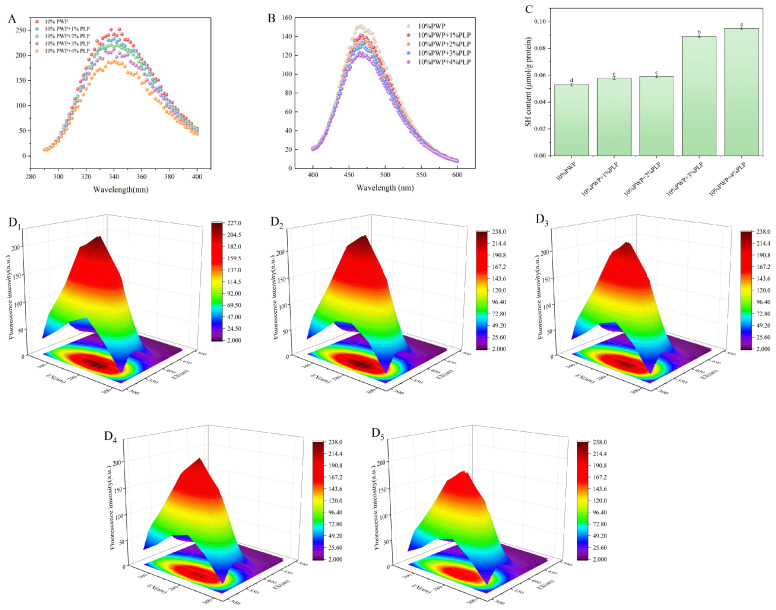
(**A**) 2D intrinsic fluorescence spectra, (**B**) surface hydrophobicity index, (**C**) free thiol density, and (**D_1_**–**D_5_**) 3D fluorescence landscapes for the PWP-PLP complex containing 10% (*w*/*w*) PWP.

The 2D and 3D intrinsic fluorescence spectra of PWP-PLP hydrogels are shown in Figure 2(A,D_1_–D_5_). The PLP0 sample exhibited a typical emission peak at approximately 345 nm, reflecting the native conformation of PWP, in which tryptophan residues remain partially exposed to a hydrophobic microenvironment [43]. As the PLP level increased, the fluorescence intensity progressively decreased, indicating a reduction in the exposure of aromatic amino acid residues. This quenching effect suggested that protein–polysaccharide interactions might encapsulate these residues within the hydrogel network [8]. The fluorescence quenching observed in the PWP-PLP system was consistent with previous findings on protein–polysaccharide complexes. For instance, in WPI–chia seed gum composites, a similar decline in fluorescence intensity was attributed to the formation of hydrogen bonds and electrostatic interactions that stabilized the protein structure and reduced solvent exposure of aromatic residues [43]. In the present study, the addition of PLP likely promoted intermolecular cross-linking within PWP, leading to a more ordered tertiary structure. The reduced accessibility of hydrophobic residues indicates the formation of a denser network, which might contribute to improved WHC and enhanced mechanical properties. Such structural rearrangements were critical for reinforcing hydrogel stability [44,45].

### 3.7. Surface Hydrophobicity (H_0_) Analysis

As shown in Figure 2B, the surface hydrophobicity (H_0_) of PWP-PLP hydrogels decreased progressively with increasing PLP level. The PLP0 sample exhibited the highest H_0_ value, indicating extensive exposure of hydrophobic residues on the protein surface. Upon the addition of PLP1, H_0_ showed a slight reduction, suggesting that PLP molecules partially shielded hydrophobic groups. In the PLP2, PLP3, and PLP4 samples, H_0_ continued to decline, reflecting the gradual decrease in hydrophobic surface exposure. This trend aligned with previous findings in other protein–polysaccharide systems, where the introduction of *Mesona chinensis* polysaccharide or *fucoidan* similarly reduced surface hydrophobicity by interacting with hydrophobic protein domains [8,9,46]. The reduction in surface hydrophobicity could be attributed to the hydrophilic nature of PLP. The abundant hydroxyl and carboxyl groups in PLP might form hydrogen bonds or electrostatic interactions with polar regions of PWP, promoting the burial of hydrophobic residues within the hydrogel matrix [35]. Additionally, enhanced hydration induced by PLP might improve the solubility of the protein–polysaccharide complexes, diminishing the tendency of hydrophobic groups to migrate toward the surface [47]. Such structural rearrangements were crucial for stabilizing the hydrogel network, as reduced surface hydrophobicity could minimize nonspecific protein aggregation and facilitate more ordered interactions between PWP and PLP.

### 3.8. Free Sulfhydryl Groups

The free sulfhydryl (SH) content of PWP-PLP hydrogels is shown in Figure 2C (Different letters indicate significant differences). As the PLP level increased from 1% to 4%, the SH levels rose significantly (*p* < 0.05). At the initial stage of heat induction, PWP molecules exhibited a tightly folded conformation, and the hydrophobic sulfhydryl groups were buried in the internal hydrophobic core of the molecules, making them undetectable. When PWP was subjected to heating, its native structure denatured, and the originally buried sulfhydryl groups began to be exposed [10,35]. The addition of PLP further promoted this process: the hydroxyl and carboxyl groups on PLP molecules could bind to PWP through hydrogen bonding and hydrophobic interactions, disrupting the weak intramolecular interactions (e.g., hydrogen bonds and hydrophobic bonds) of PWP. This effect synergized with heating to facilitate the unfolding and stretching of protein peptide chains, thereby increasing the accessibility of sulfhydryl groups [8]. This was the core reason why the increase in PLP level led to a significant rise in free sulfhydryl content. Such structural rearrangement of protein molecules is crucial for the formation of disulfide bonds that stabilize the hydrogel network [9]. The sulfhydryl groups with improved accessibility provide abundant reactive sites for the oxidative formation of intermolecular disulfide bonds. As covalent cross-linking bridges, disulfide bonds can interconnect dispersed PWP molecules and PWP-PLP complexes, constructing a more compact, homogeneous, and robust three-dimensional hydrogel network. This optimized network structure can effectively reduce the formation of large pore defects and structural collapse, thereby significantly enhancing the water-holding capacity, mechanical strength, elasticity, and storage stability of the hydrogels [48].

### 3.9. Simultaneous Rheology (SR) and Fourier Transform Infrared (FTIR) Spectroscopy (SR-IR)

**Figure 3:**
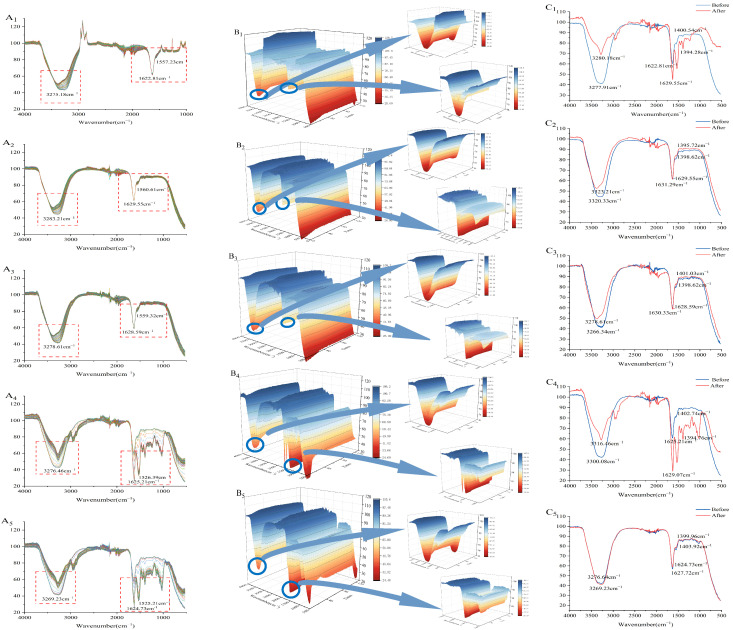
2D FTIR profiles (**A_1_**–**A_5_**), 3D FTIR landscapes (**B_1_**–**B_5_**), and comparative 2D FTIR traces of the starting and endpoint states (**C_1_**–**C_5_**) for PWP-PLP complexes containing 10% (*w*/*w*) PWP and graded PLP concentrations (0%, 1%, 2%, 3%, and 4% *w*/*v*). Boxes on the left denote Amide A, those on the right denote Amide I.

During the formation of PWP-PLP hydrogels, intermolecular interactions played a central role in determining the hydrogel network’s architecture and its structural stability. Fourier transform infrared spectroscopy (FTIR), which reflects the vibrational behavior of characteristic functional groups, was employed to elucidate the interaction mechanisms between PWP and PLP. Combined with rheological measurements, these data further clarify how varying PLP levels regulate the structure of the composite hydrogel. As shown in Figure 3(A_1_–B_5_), each spectrum corresponds to a PWP-PLP hydrogel with different PLP addition levels. In the region of 3200–3600 cm^−1^, the -OH stretching band in the PLP0 system exhibited a relatively sharp profile, whereas the peak became progressively broader with the incorporation of PLP. This broadening suggested the establishment of new hydrogen bond interactions between PWP and PLP, leading to a reorganization of the original supramolecular network. Similar hydrogen bond-mediated structural adjustments have been reported in other protein–polysaccharide systems [44]. Across PLP addition levels (0%, 1%, 2%, 3% and 4%), the widening of the -OH band displayed a clear level-dependent trend, indicating that higher PLP contents provided more hydroxyl and carboxyl groups capable of forming dense hydrogen bonding and electrostatic interaction sites with PWP. This enhanced molecular association contributes to a more compact and integrated hydrogel network. Notably, characteristic changes were also observed in the amide I (1600–1700 cm^−1^) and amide II (1500–1600 cm^−1^) regions. Increasing PLP levels induced a redshift in the amide I band in certain samples, suggesting strengthened hydrogen bonding and a transition of PWP secondary structures toward more ordered and compact β-sheet conformations. In contrast, a slight blueshift observed at low PLP levels implies weakened hydrogen bonding and partial relaxation or rearrangement of peptide segments. Such red-/blueshift phenomena have been widely reported in protein–polysaccharide complexes and are regarded as spectral indicators of polysaccharide-induced conformational modulation [49,50]. These FTIR observations align well with the rheological results, where enhanced gelation kinetics and network rigidity were detected at higher PLP levels, underscoring the cooperative role of PLP in stabilizing the protein molecular framework and promoting hydrogel structuring.

Based on the initial and final SR-IR spectra (Figure 3(C_1_–C_5_)), thermal induction further affected the PWP-PLP system. The amide A band shifted from 3277.91, 3320.33, 3266.34, 3300.08, and 3269.23 cm^−1^ to 3280.18, 3323.21, 3278.61, 3316.46, and 3276.64 cm^−1^, respectively. The overall blueshift indicated modifications in the secondary structure, likely due to disruption of conjugation and reorganization of hydrogen bonding during thermal gelation, resulting in altered spectral absorption behavior [8].

### 3.10. Two-Dimensional Correlation Spectroscopy (2D-COS)

**Figure 4:**
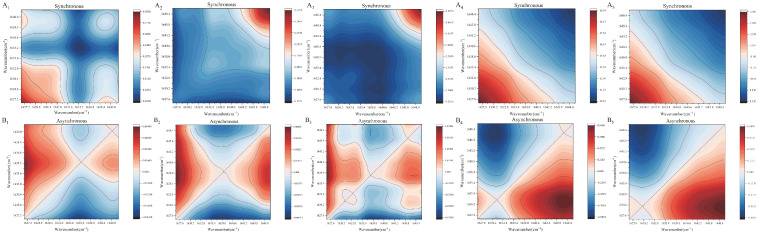
2D-COS analyses of PWP-PLP systems (10% *w*/*w* PWP) at varying PLP concentrations (0%, 1%, 2%, 3%, and 4% *w*/*v*): synchronous maps (**A_1_**–**A_5_**) and asynchronous maps (**B_1_**–**B_5_**).

2D-COS was employed to further elucidate the molecular interactions and structural evolution of PWP-PLP hydrogels under thermal perturbation. Compared with conventional FTIR, 2D-COS provides enhanced spectral resolution and allows for sequential analysis of functional group responses to external stimuli [51]. As shown in Figure 4(A_1_–B_5_), the synchronous spectra exhibited a pronounced autocorrelation peak near 1630 cm^−1^, which was attributed to the C=O stretching vibration in the amide I region, indicating that this group was highly sensitive to thermal treatment and played a key role in hydrogel network formation. In the asynchronous spectra, clear off-diagonal cross-peaks appeared between 1628–1635 cm^−1^ and 1638–1647 cm^−1^, suggesting that the responses of C=O stretching vibrations were not synchronized with those of O-H and C-O modes during heating. This asynchronous behavior implied a sequential rearrangement of hydrogen bonding and protein–polysaccharide interactions rather than a simultaneous structural transition [51]. Notably, the intensity of these cross-peaks increased with increasing PLP level, indicating that higher PLP levels promoted stronger intermolecular interactions, likely due to the abundant hydroxyl and carboxyl groups facilitating hydrogen bond formation with PWP [51,52]. Additionally, SR-IR analysis revealed a blueshift in the amide A band after thermal induction, suggesting disruption of the original hydrogen-bonded conjugated system and subsequent reorganization of protein secondary structures [53]. These molecular-level changes were consistent with the enhanced rheological properties observed for PWP-PLP hydrogels, confirming that PLP effectively regulates protein conformational rearrangement and contributes to the formation of a more stable and ordered hydrogel network.

### 3.11. Molecular Docking

**Figure 5:**
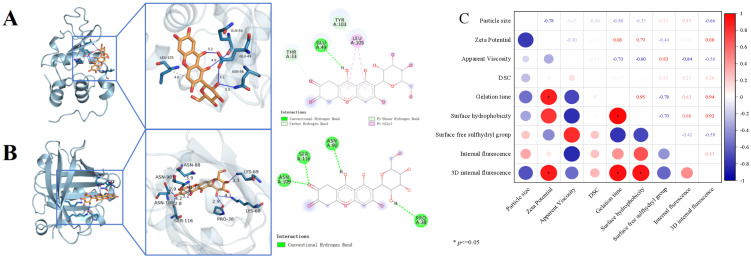
Molecular docking of α-lactalbumin-PLP (**A**), β-lactoglobulin-PLP (**B**), and correlation matrix for the PWP-PLP complex containing 10% (*w*/*w*) PWP (**C**).

Figure 5A,B depict the interactions of PLP with α-lactalbumin (A) and β-lactoglobulin (B), with their respective binding energies quantified as −6.5 kcal/mol and −6.7 kcal/mol. For ligand-target binding, a value below 0 kcal/mol is indicative of spontaneous association between the ligand and the target protein, while a binding energy more negative than −5.0 kcal/mol denotes robust affinity between the active small-molecule component and the target, which in turn confers stable binding between the small molecule and the protein [54]. In the 3D interaction visualizations, hydrogen bonds are marked with blue solid lines, and hydrophobic forces are indicated by gray solid lines. In the 2D complex structure maps, green and light green dashed lines are both assigned to represent intermolecular hydrogen bonds, while pink dashed lines designate hydrophobic interactions [25].

Figure 5A depicts the binding energy between the small molecule PLP and α-lactalbumin as −6.5 kcal/mol. Specifically, PLP forms two hydrogen bonds (3.1 Å and 3.2 Å in length, respectively) with the GLU49 residue of α-lactalbumin, and an additional two hydrogen bonds (3.3 Å and 4.0 Å) that interact with the ASN56 and GLN54 residues of the same protein. Furthermore, a hydrophobic interaction (4.1 Å) mediates the binding of PLP to the LEU105 residue. Consistent results were obtained from the 2D interaction analysis, and the synergistic effects of these intermolecular forces enable the stable binding of PLP to α-lactalbumin.

Figure 5B illustrates that the binding energy of PLP with β-lactoglobulin is −6.7 kcal/mol. PLP interacts with β-lactoglobulin through multiple intermolecular forces: three hydrogen bonds (2.9 Å, 3.3 Å, and 4.1 Å) that target the PRO38, LYS60, and LYS69 residues, respectively; two hydrogen bonds (3.2 Å and 3.7 Å) that bind to the ASN90 residue; a hydrogen bond (2.9 Å) and a hydrophobic interaction (4.2 Å) that link to the ASN109 residue; and a hydrogen bond (2.8 Å) and a hydrophobic interaction (3.9 Å) that associate with the SER116 and ASN88 residues, respectively. Parallel findings were observed in the 2D interaction profiling, and these collective intermolecular interactions facilitate the stable binding of PLP to the receptor β-lactoglobulin.

### 3.12. Correlation Analysis

Correlation analysis enables the evaluation of the overall correlative characteristics of samples and facilitates the exploration of relationships between the physicochemical properties and structural changes to the tested samples [14]. Figure 5C intuitively illustrates the association patterns among nine physicochemical and structural indices: red indicates a positive correlation, while blue denotes a negative correlation, and the absolute value of the correlation coefficient, combined with significance markers, reflects the strength of the association. The results show that gelation time exhibited an extremely strong positive correlation with zeta potential, surface hydrophobicity, and 3D internal fluorescence. In contrast, apparent viscosity and surface free sulfhydryl groups were strongly negatively correlated with gelation time, indicating that a decrease in the former was accompanied by an extension of gelation time. These findings suggest that, as the PLP level increases, hydrophobic interactions, hydrogen bonds, and disulfide bonds promote the formation of a larger and more stable gel matrix in PWP-PLP, which improves the tertiary structure of PWP and thus facilitates the application of the PWP-PLP hydrogel system in yogurt.

### 3.13. pH Analysis of Yogurt with Added PWP-PLP

**Figure 6:**
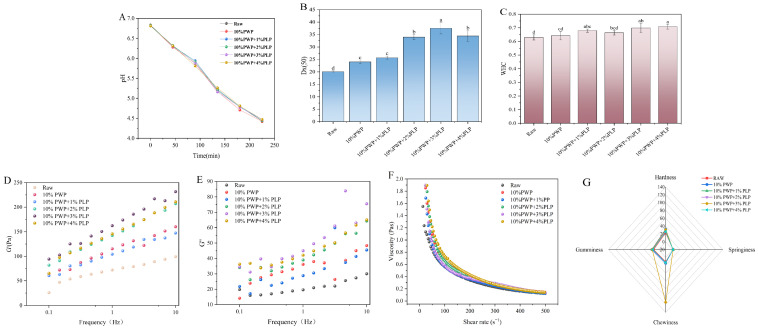
pH (**A**), size (**B**), WHC (**C**), G′ (**D**), G″ (**E**), apparent viscosity (**F**), and texture (**G**) of yogurt.

During yogurt fermentation, pH was monitored at regular intervals as an indicator of lactic acid production and fermentation progress. The fermentation was terminated by rapid cooling when the pH reached approximately 4.5. Under simulated processing and storage conditions, the pH stability of five yogurt formulations was monitored: a blank control (fermented yogurt without PLP), a formulation containing 10% PWP alone, and 10% PWP formulations supplemented with 1%, 2%, 3%, or 4% PLP (Figure 6A). All samples exhibited a gradual decrease in pH over time, reflecting the inherent acid–base instability of yogurt systems. Although the initial pH values (t = 0) were comparable among all groups (*p* > 0.05), PLP-containing formulations showed superior pH stability, with significantly lower rates of pH decline than the control (*p* < 0.05). Notably, the yogurt containing 3% PLP maintained the highest pH stability throughout storage, indicating a level-dependent ability of PLP to maintain acid–base balance through its interactions with protein and aqueous components. These findings supported the potential of PLP as an effective natural stabilizer to mitigate pH fluctuations [14].

### 3.14. Particle Size Analysis of Yogurt with Added PWP-PLP

Particle size critically influences the mouthfeel and stability of yogurt [52]. Upon the incorporation of the PWP-PLP hydrogel, the average particle size (Dx50) of yogurt increased and reached a maximum at a PLP level of 2%, followed by a subsequent decrease (Figure 6B) (Different letters indicate significant differences). This behavior might be attributed to electrostatic interactions and hydrogen bonding between PLP molecules, denatured whey proteins, and casein micelles at low PLP levels, which promoted protein aggregation and bridging, thereby forming a denser hydrogel network with larger particle sizes [55]. As the PLP level continued to increase, the steric hindrance and water-binding capacity of the polysaccharide became more pronounced, inhibiting excessive protein aggregation and leading to a more homogeneous hydrogel structure, which in turn resulted in reduced particle sizes [56]. An optimal particle size distribution contributed to a smoother and creamier yogurt texture with less perceived graininess, while simultaneously enhancing physical stability and reducing serum (whey) separation.

### 3.15. Analysis of WHC of Goat Yogurt with PWP-PLP

WHC is a critical indicator of yogurt quality and stability, as it directly affects texture, shelf life, and consumer acceptance [57]. PLP, as a natural bioactive component, could modulate the microstructure of the yogurt system and thereby influence water distribution and retention. As shown in Figure 6C (Different letters indicate significant differences), the WHC of yogurt increased progressively with increasing PLP level. The blank and 0% PLP groups exhibited relatively low WHC values, indicating that, in the absence of PLP, the yogurt matrix had limited water-retention capacity and was prone to texture deterioration dude to serum loss. In contrast, the low-level PLP treatments (1%, 2%, and 3% PLP) showed improved WHC, suggesting that even low levels of PLP could interact with milk proteins to optimize the water-holding network preliminarily. The 4% PLP group exhibited the highest WHC among all formulations, implying that a higher PLP level could establish a more effective water-retention structure. Overall, the WHC measurements clearly demonstrated that PLP could significantly enhance the WHC of yogurt, with a level-dependent improvement. Owing to its hydrophilic functional groups and intermolecular interactions, PLP contributed to the formation of a more stable water-holding network, thereby fundamentally improving moisture retention in yogurt [19,58].

### 3.16. Rheological Properties of Yogurt

#### 3.16.1. Analysis of G′ and G″ Moduli of Yogurt Containing PWP-PLP

The rheological properties of the yogurt samples were characterized in terms of G′ and G″. As shown in Figure 6D,E, the overall distribution range of these parameters shifted upward with increasing PLP level, indicating that the incorporation of PWP-PLP hydrogels promoted the formation of a more stable and compacted network structure in the yogurt matrix, thereby enhancing its structural stability. This could be attributed to the formation of additional hydrogen bonds and other intermolecular interactions between the PWP-PLP colloidal network and casein micelles, which strengthened the connections between them. At the same time, hydrophobic interactions favored the formation of larger protein aggregates, contributing to a more continuous hydrogel network and a tighter packing of protein clusters [19,29,59]. Consequently, the texture of the yogurt was improved, resulting in a smoother product that is easier to swallow.

#### 3.16.2. Apparent Viscosity of the PWP-PLP Yogurt System

Apparent viscosity is a key indicator of yogurt texture and mouthfeel, directly affecting both consumer acceptance and product stability. PLP may modulate the rheological behavior of yogurt systems through intermolecular interactions and thereby alter their viscosity characteristics. As shown in Figure 6F, apparent viscosity decreased with increasing PLP level; however, the rate of decline, the final steady-state viscosity, and the level-dependent response differed markedly among the formulations. In the low-PLP samples (1–2%), the limited amount of PLP was insufficient to extensively cross-link with milk proteins and to build a continuous thickening network. Instead, the small quantity of PLP likely interfered with the spontaneous aggregation of milk proteins and disrupted the primary viscosity-building structure, resulting in a lower initial viscosity and a weak response to shear, ultimately leading to a low-viscosity steady state [60]. At higher PLP levels (3–4%), PLP molecules interacted with milk proteins via hydrogen bonding and hydrophobic interactions, forming a denser three-dimensional network. This network effectively immobilized water molecules and enhanced internal friction and resistance to shear. As the number of cross-linking points and the compactness of the network increased, its contribution to viscosity and viscosity stability was strengthened. Overall, these results indicated a positive relationship between PLP level and viscosity stability, providing a theoretical basis for the incorporation of PLP into yogurt formulations and supporting its potential in the development of innovative functional yogurts.

### 3.17. Texture Analysis of Yogurt

Texture is one of the key determinants of yogurt’s sensory quality, consumer acceptance, and product stability [52]. In this study, four commonly used textural parameters—hardness, gumminess, springiness, and chewiness—were measured, and the results are shown in Figure 6G. As the PLP level increased, these parameters initially increased and then decreased. When the PLP level was below 3%, all textural attributes gradually increased, indicating that an appropriate amount of PLP could interact with milk proteins through electrostatic forces and hydrogen bonding to form composite network structures. Such interactions promoted protein cross-linking and gelation, leading to a denser and more stable three-dimensional hydrogel matrix in yogurt [52]. Among all samples, the yogurt containing 10% PWP + 3% PLP exhibited the highest hardness, elasticity, adhesiveness, and chewiness values, suggesting that this level enabled PLP to interact most effectively with milk proteins. This enhanced the integrity and resilience of the hydrogel network, thereby improving the yogurt’s thickness and smoothness. However, when the PLP level further increased to 4%, the textural parameters slightly decreased. This decline might be attributed to excessive polysaccharides introducing steric hindrance and inducing local phase separation within the system, which weakens protein–protein interactions and results in an overly compact but less flexible hydrogel network [61]. Overall, the addition of an appropriate amount of PLP (approximately 3%) helped optimize both the microstructure and macroscopic textural properties of the yogurt, ultimately enhancing product stability and sensory quality.

### 3.18. Microstructure of Goat Yogurt with PWP-PLP

**Figure 7:**
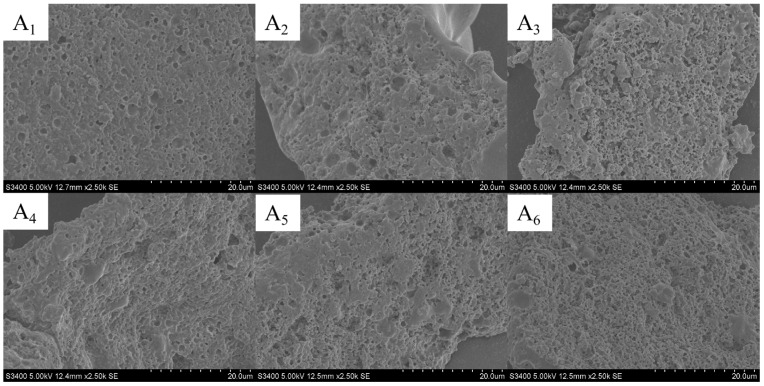
SEM ((**A_1_**–**A_6_**), ×2.5 k) of yogurt using PWP-PLP as a food additive.

The microstructure of yogurt is a fundamental factor determining its texture, stability, and functional properties [62]. PLPs, as natural bioactive polysaccharides, could interact with milk proteins in the yogurt matrix, analogous to the thermo-induced gelation behavior of PWP—thereby reconstructing the microstructural network. In this study, scanning electron microscopy (SEM) was employed to investigate the effects of different PLP levels on the microstructure of yogurt (Figure 7(A_1_–A_6_)). The results showed that the control sample with no PWP-PLP addition exhibited a relatively loose microstructure with unevenly distributed pores, characterized by large voids and regions of protein aggregation. These features indicated structural defects arising from spontaneous protein aggregation, which were prone to causing whey separation and poor textural stability. As the PLP level increased, the yogurt hydrogel network gradually became more compact. In the 1% and 2% PLP groups, the pore size visibly decreased, and the pore distribution became more uniform, with improved continuity of the supporting framework, suggesting that initial interactions between PLP and milk proteins had been established. In the 3% PLP group, the pores were further refined, and the three-dimensional network appeared more ordered and tightly interconnected, reflecting a synergistic effect between PLP and milk proteins that effectively strengthened the hydrogel matrix and suppressed phase separation. When the PLP level reached 4%, the yogurt microstructure displayed an even higher degree of compactness and uniformity, featuring smaller pores and a highly stable three-dimensional supporting network. Overall, PWP-PLP composite hydrogels could be incorporated into yogurt. An elevated PLP level enhanced the electrostatic and hydrophobic interactions between PLP and milk proteins, which continuously reduced the pore size of the yogurt hydrogel network and further constructed a denser, more ordered structure [63]. These structural improvements significantly contributed to the enhanced microstructural integrity and stability of the yogurt.

## 4. Conclusions

In conclusion, this study demonstrates that *Phellinus linteus* polysaccharide (PLP) can effectively interact with heat-induced polymerized whey protein (PWP) to form a composite hydrogel with enhanced structural and functional properties. The interactions, primarily driven by hydrogen bonding and hydrophobic forces, led to increased colloidal and thermal stability, a more compact network, and higher water-holding capacity. When incorporated into goat milk yogurt, the optimized PWP-PLP composite (with 3% PLP) served as an excellent texture modifier, significantly improving the product’s water retention, rheological properties, and microstructure, resulting in superior firmness and smoothness. These findings provide not only a deeper mechanistic understanding of PLP-PWP interactions but also a practical, natural strategy for developing high-quality yogurt, particularly with potential applications in formulating specialized nutritional products dysphagia-friendly foods. Future research could explore the long-term stability and sensory acceptance of PWP-PLP-fortified yogurt, investigate the in vitro bioaccessibility of bioactive components, and extend this protein–polysaccharide design strategy to other dairy or plant-based matrices for developing novel functional foods.

## Data Availability

The original contributions presented in this study are included in the article. Further inquiries can be directed to the corresponding authors.
